# Enhanced Specificity in Loop-Mediated Isothermal Amplification with Poly(ethylene glycol)-Engrafted Graphene Oxide for Detection of Viral Genes

**DOI:** 10.3390/bios12080661

**Published:** 2022-08-20

**Authors:** Jamin Ku, Khushbu Chauhan, Sang-Hyun Hwang, Yong-Joo Jeong, Dong-Eun Kim

**Affiliations:** 1 Department of Bioscience and Biotechnology, Konkuk University, 120 Neungdong-ro, Gwangjin-gu, Seoul 05902, Korea; 2 Department of Laboratory Medicine, Asan Medical Center, University of Ulsan College of Medicine, Seoul 05505, Korea; 3 School of Applied Chemistry, Kookmin University, Seoul 02707, Korea

**Keywords:** loop-mediated isothermal amplification (LAMP), non-specific DNA amplification, poly(ethylene glycol) engrafted nanosized GO (PEG-nGO), hepatitis C virus (HCV)

## Abstract

Loop-mediated isothermal amplification (LAMP) is a nucleic acid amplification method that allows the simple, quick, and low-cost detection of various viral genes. LAMP assays are susceptible to generating non-specific amplicons, as high concentrations of DNA primers can give rise to primer dimerization and mismatched hybridizations, resulting in false-positive signals. Herein, we reported that poly(ethylene glycol)-engrafted nanosized graphene oxide (PEG-nGO) can significantly enhance the specificity of LAMP, owing to its ability to adsorb single-stranded DNA (ssDNA). By adsorbing surplus ssDNA primers, PEG-nGO minimizes the non-specific annealing of ssDNAs, including erroneous priming and primer dimerization, leading to the enhanced specificity of LAMP. The detection of complementary DNAs transcribed from the hepatitis C virus (HCV) RNA was performed by the PEG-nGO-based LAMP. We observed that the inclusion of PEG-nGO significantly enhances the specificity and sensitivity of the LAMP assay through the augmented difference in fluorescence signals between the target and non-target samples. The PEG-nGO-based LAMP assay greatly facilitates the detection of HCV-positive clinical samples, with superior precision to the conventional quantitative real-time PCR (RT-qPCR). Among the 20 clinical samples tested, all 10 HCV-positive samples are detected as positive in the PEG-nGO-based LAMP, while only 7 samples are detected as HCV-positive in the RT-qPCR. In addition, the PEG-nGO-based LAMP method significantly improves the detection precision for the false-positive decision by 1.75-fold as compared to the LAMP without PEG-nGO. Thus, PEG-nGO can significantly improve the performance of LAMP assays by facilitating the specific amplification of target DNA with a decrease in background signal.

## 1. Introduction

Loop-mediated isothermal amplification (LAMP), as one of the isothermal nucleic acid amplification methods, attracted a great amount of attention in molecular diagnostics [1,2,3,4]. Since LAMP is conducted under isothermal conditions, without the need of laborious thermal cycling, it has been widely adapted as a rapid and robust method for nucleic acid amplification at a constant temperature (60 to 65 °C) within 1 h [2,5,6]. The underlying mechanism for isothermal DNA amplification with LAMP is to use DNA primers to generate foldback structures, and their subsequent extension with *Bacillus stearothermophilus* (*Bst*) DNA polymerase, through strand displacement activity [7,8,9]. Amplified double-stranded DNAs (dsDNA) are continuously monitored by using DNA-staining dyes, such as SYBR Green or SYTO, during the LAMP reaction through fluorescence enhancement owing to the intercalation of dyes in dsDNA [10,11,12]. However, LAMP reactions are prone to often producing undesirable non-specific amplification products as a consequence of primer dimerization and mismatched hybridization, because LAMP assays need four to six DNA primers at high concentrations (3.6–4.4 μM) as compared to two DNA primers (0.4–1.0 μM) employed in a conventional polymerase chain reaction (PCR) [13,14,15]. Since fluorescence enhancement is attributed to amplicon DNAs in the LAMP reaction, the fluorescence signal cannot distinguish between target-specific amplicon DNA from false-positively amplified DNA [16]. In particular, primer dimerization between inner primers, which usually contains 40 to 50 bps with the highest concentrations among primer DNAs used in LAMP, often leads to background fluorescence enhancement through the accumulation of non-specifically amplified DNAs intercalated with staining dye [14,17,18].

To improve the specificity of LAMP, by a reduction in background fluorescence and an increase in signal fluorescence, a variety of approaches have been suggested, such as designing strand-displacement probes [10], carboxamide and *N*-alkylcarboxamide [19], pullulan [20], guanidine chloride [21], touchdown temperature with additives such as dimethyl sulfoxide [22], and a Janus probe system [13]. In addition, gold nanoparticles [23] were used to exert a hot-start effect in the LAMP, and single-strand DNA binding (SSB) protein [24] was employed to suppress the interaction between template DNAs. Recently, a graphene oxide (GO)-based LAMP method was developed to enhance the specificity of LAMP, with a wider dynamic range to distinguish true from false amplicons [14]. GO as a two-dimensional (2D) nanomaterial with a high surface area [25,26] was used to reduce the non-specific hybridization of primer DNAs, because GO tends to adsorb only single-stranded nucleic acid (ssDNA and RNA) rather than double-stranded DNA [14,27,28]. However, the use of GO in nucleic acid amplification reactions such as PCR and LAMP is often challenging with the adverse features of GO, including low dispersibility in the presence of divalent cation (i.e., Mg^2+^) [29,30] and protein adsorption onto the GO surface, which causes protein aggregation with a loss in enzyme function [31,32,33]. GO was used to adsorb ssDNA primers for specificity enhancement in PCRs by lowering background signal caused by non-specific DNA amplification [34,35]. GO decreases the non-specific annealing of ssDNA, such as primer dimerization and false priming, by adsorbing excess primers during a PCR. However, DNA polymerase, which is essential in nucleic acid amplification, tends to be adsorbed to the GO surface through non-covalent bonds, displaying reduced function with low efficacy in nucleic acid amplification.

We previously fabricated a GO surface with a biocompatible polymer such as poly(ethylene glycol) (PEG), in order to enhance the dispersibility of GO in solutions with high salt concentration in the PCR mixture, and to minimize non-specific protein adsorption without hampering the properties of GO as a ssDNA-adsorbing material [29]. This poly(ethylene glycol)-engrafted nanosized GO (PEG-nGO) demonstrates the enhancement of PCR specificity by preferential binding to ssDNA, without adsorbing DNA polymerase.

Encouraged by the superb efficiency of PEG-nGO in PCR, we explored the hypothesis that PEG-nGO in LAMP would retain the beneficial properties of GO as an adsorbent of ssDNA and facilitate LAMP reactions containing high concentrations of several ssDNA primers. We employed PEG-nGO in the LAMP reaction for the detection of the hepatitis C virus (HCV) gene as a model system. We observed that the addition of PEG-nGO in the LAMP prevents the non-specific amplification by primer dimerization through the adsorption of the excess amount of ssDNA primers with a reduction in high background fluorescence signal. In addition, the developed PEG-nGO-based LAMP was successfully employed to detect HCV-positive clinical samples, providing a practical perspective in the LAMP-based nucleic acid detection.

## 2. Materials and Methods

### 2.1. Chemical Reagents and DNA Polymerase

GO was purchased from Graphene Supermarket (Calverton, NY, USA) and 6-arm poly(ethylene glycol)-amine (15 kDa) was purchased from SunBio (Seoul, Korea). *N*-3-(dimethylamino)propyl-*N*′-ethylcarbodiimide hydrochloride (EDC) and chloroacetic acid were purchased from Sigma-Aldrich (St. Louis, MO, USA). 2-mercaptoethanol and sodium hydroxide were purchased from Bio Basic Inc (Ontario, Canada). 10× isothermal amplification buffer II, 100 mM MgSO_4_, and *Bst* 3.0 DNA polymerase were purchased from New England Biolabs (Ipswich, MA, USA). Deoxynucleotides (dATP, dTTP, dGTP, dCTP: 100 mM each), and SYBR^™^ Green I were purchased from Invitrogen (Carlsbad, CA, USA). GelRed^®^ nucleic acid gel stain was purchased from Biotium (Fremont, CA, USA).

### 2.2. Primer DNAs

LAMP primers and RT-qPCR primers were designed based on hepatitis C virus genotype 1, complete genome (GenBank accession NC_004102). The open source software New England Biolabs (NEB) LAMP primer design tool (https://lamp.neb.com (accessed on 22 March 2022)) with default parameters was used to design suitable LAMP primer set. Sequences of DNA primers for RT-qPCR (forward and reverse) and LAMP DNA primer set comprising of two outer primers (F3/B3), two inner primers (FIP/BIP), and two loop primers (LF/LB) are listed in Table S1. DNA oligonucleotides used for gene amplification (LAMP and RT-qPCR) were chemically synthesized and purified by high affinity purification (Bionics, Seoul, Korea).

### 2.3. Total RNA Extraction and Reverse Transcription

Total HCV RNA was extracted from human serum samples obtained from Asan Medical Center (Seoul, Korea), with an approval of the internal review board (IRB No. 2017-1162). Viral RNA extraction was performed using the QIAamp^®^ Viral RNA Mini kit (Qiagen, Hilden, Germany), according to the manufacturer’s protocol. Extracted RNA concentrations were quantified using a UV/Visible spectrophotometer (Ultrospec 2100 pro spectrophotometer: Biochrom Ltd., Cambridge, UK). Extracted viral RNAs were reverse-transcribed to synthesize complementary DNA (cDNA) using the ReverTra Ace^®^ qPCR RT Master Mix kit (Toyobo, Osaka, Japan). The cDNA samples were stored at −20 °C until further use.

### 2.4. Preparation of PEG-nGO

PEG-nGO was prepared according to previous protocol of our laboratory [29]. Briefly, GO (2 mg mL^−1^) was cracked into the nanosized GO (nGO) by tip sonication for 5 h on an ice bath. Sodium hydroxide (0.24 g mL^−1^) and chloroacetic acid (0.2 g mL^−1^) were added to the nGO suspension and bath sonicated for 3 h to convert -OH groups to -COOH groups, resulting in carboxylated nGO (nGO–COOH). nGO–COOH solution was purified by repeated rinsing and filtrations with distilled water, using 0.2 µm membrane filter (Millipore, Billerica, MA, USA). The filtered nGO–COOH solution was diluted by distilled water to give an optical density of 0.4 at 808 nm. The 6-arm PEG-amine (2 mg mL^−1^) was added to the filtered nGO–COOH solution and bath sonicated for 10 min. EDC (5 mM) was added to the nGO–COOH solution and bath sonicated for 5 min, with subsequent stirring for 12 h at 25 °C. The EDC coupling reaction was terminated by adding 2-mercaptoethanol (50 mM), and the solution was centrifuged at 10,000× *g* for 1 h in phosphate-buffered saline. The resulting supernatant containing PEG-nGO was stored at 4 °C for subsequent use.

### 2.5. LAMP Assays

LAMP assays with or without PEG-nGO were designed to amplify the target HCV cDNA. The LAMP reaction with PEG-nGO in a 25 μL reaction mixture contained 1× isothermal amplification buffer II (20 mM Tris-HCl, 150 mM KCl, 10 mM (NH_4_)_2_SO_4_, 2 mM MgSO_4_, 0.1% (*v/v*) Tween 20, pH 8.8), 1.4 mM dNTP mixture, 6 mM MgSO_4_, LAMP primer mixture (0.2 µM F3/B3, 1.6 µM FIP/BIP, and 0.4 µM Loop F/B), *Bst* 3.0 DNA polymerase (8 U), 1× SYBR Green I, and 1 μL of HCV cDNA sample or doubled-distilled water (i.e., blank) in the presence of varied concentration of PEG-nGO (0 to 15 μg mL^−1^). The LAMP reactions were performed in a real-time thermocycler Rotor Gene Q system (Qiagen, Hilden, Germany), with real-time fluorescence monitoring at 60 °C for 60 min followed by 5 min incubation at 72 °C for reaction termination.

### 2.6. Gel Electrophoresis

The amplified DNA products of LAMP reactions with or without PEG-nGO were analyzed by gel electrophoresis for the amplified DNAs. Amplified DNAs after the LAMP reaction was resolved in 2% agarose gel in 1× TAE buffer at 100 V for 40 min, and stained with GelRed^®^ stain.

### 2.7. ssDNA Adsorption on PEG-nGO

ssDNA (50 nM, 40-mer) labeled with 5′-carboxyfluorescein (FAM–DNA) was mixed with increasing concentrations (0, 0.5, 1, 5, 10, 15, 20, 30, and 40 μg mL^−1^) of PEG-nGO, and incubated at 60 °C for 10 min. Fluorescence intensity was measured at an excitation wavelength of 485 nM and emission wavelength at 535 nM by a multilabel plate reader (VICTOR X3; PerkinElmer, Waltham, MA, USA).

### 2.8. RT-qPCR

HCV viral RNA was extracted from HCV patients’ serum samples by QIAamp^®^ Viral RNA Mini kit, and then transcribed into cDNA using ReverTra Ace^®^ qPCR RT Master Mix, according to the manufacturer’s protocol. RT-qPCR was performed with cDNA using the QuantiNova^®^ SYBR^®^ Green PCR kit (Qiagen) in a 20 μL reaction mixture containing 1× QuantiNova SYBR Green PCR master mix (Qiagen), 1 μL of cDNA, and PCR primers (200 nM) in a real-time thermocycler Rotor Gene Q system (Qiagen) under the following conditions; pre-denaturation at 95 °C for 2 min, followed by 40 cycles of 30 s at 95 °C, 30 s at 57 °C, and 30 s at 68 °C, and a final extension at 68 °C for 5 min.

## 3. Results and Discussion

### 3.1. Working Mechanism of LAMP Facilitation by PEG-nGO

**Scheme 1:**
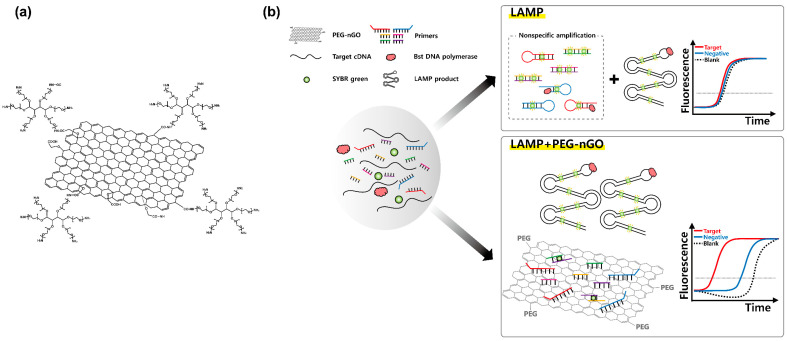
Schematic illustration of loop-mediated isothermal amplification (LAMP) facilitation by poly(ethylene glycol)-engrafted nanosized graphene oxide (PEG-nGO). (**a**) Expected structure of PEG-nGO. (**b**) Schematic diagram showing the effect of PEG-nGO on LAMP specificity.

The possible working mechanism of the facilitation of LAMP by PEG-nGO in terms of enhanced specificity is shown in Scheme 1. We coated the nanosized GO (nGO) surface with PEG for the enhancement of nGO solubility in LAMP reaction mixture and unhindered polymerase activity (Scheme 1a). The utilization of high amounts of primers in a typical LAMP system makes them more prone to generate primer dimers and mismatched hybridizations, resulting in non-specific amplification and a strong background fluorescence signal. The intercalation of dsDNA binding fluorescent dye in dsDNA amplicons generates the fluorescent signal irrespective of the presence or absence of specific target amplicon DNA, leading to the generation of false-positive signals. On the contrary, we hypothesized that the inclusion of PEG-nGO in a LAMP reaction can enhance specificity, as primers would adsorb onto PEG-nGO with a substantial binding affinity due to its ssDNA binding ability. In the presence of a specific target, primers adsorbed on the PEG-nGO would desorb and hybridize with the target, owing to the stronger base pairing affinity between the primer and target than that between the primer and PEG-nGO. In the PEG-nGO-based LAMP, primers are available only in the presence of a specific target for hybridization, abolishing the false-positive signals (Scheme 1b). Thus, we investigated whether PEG-nGO can suppress the generation of non-specific amplicons by adsorbing excess primers responsible for primer dimerization and mismatched hybridization.

### 3.2. Specificity and Sensitivity of PEG-nGO-Based LAMP

**Figure 1:**
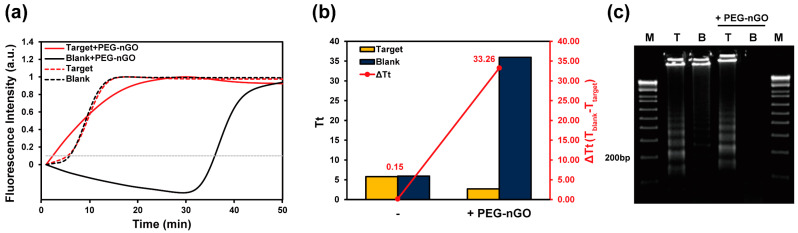
Facilitation of LAMP specificity by PEG-nGO. (**a**) Real−time fluorescence monitoring of LAMP−amplified products for the target and blank samples in the absence or presence of PEG-nGO. Horizontal dotted line at fluorescence intensity of 0.1 represents the threshold time (Tt), which is the time point at which 10% of the maximum amplified fluorescence intensity was utilized for signal quantification. (**b**) Tt values for target and blank samples in the absence or presence of PEG-nGO. ΔTt values are plotted as difference between Tt of blank sample and Tt of target sample. (**c**) Agarose gel electrophoretic analysis of the LAMP−amplified products in the absence or presence of PEG-nGO. Abbreviations: M,200 bp DNA ladders; T, specific target; B, blank (double distilled water as negative control); +PEG-nGO−LAMP in the presence of PEG-nGO.

First, to check the effect of PEG-nGO on LAMP, we carried out a LAMP specific for cDNA transcribed from HCV RNA (target) and double-distilled water as negative control (blank) in the absence or presence of PEG-nGO (Figure 1a). In the absence of PEG-nGO, the target and blank sample generate fluorescence signals with considerably similar threshold time (Tt) values, which is the time point at which 10% of the maximum amplified fluorescence intensity was utilized for signal quantification after the commencement of the LAMP reaction, resulting in the difference in Tt between the target and blank samples; ΔTt is 0.15 min (Figure 1b). On the contrary, when PEG-nGO is added to the LAMP reaction, the target-generated fluorescence signal increases much faster than the blank sample (Figure 1a), resulting in significantly increased ΔTt, as high as 33.26 min (Figure 1b). Thus, the inclusion of PEG-nGO delays fluorescence signal, with a substantial increase in Tt for the blank sample, as compared to the LAMP without PEG-nGO.

Next, to obtain the optimal concentration of PEG-nGO in the LAMP assay, various concentrations of PEG-nGO (0–15 μg mL^−1^) are used in the LAMP reaction (Figure S1). We observed that the Tt for the target sample is significantly decreased when the LAMP is supplemented with PEG-nGO (3–10 μg mL^−1^) as compared to the LAMP without PEG-nGO. Importantly, a higher concentration of PEG-nGO (15 μg mL^−1^) shows an enormous increase in Tt for the target, implying the inhibition of LAMP at higher PEG-nGO concentrations. Thus, 10 μg mL^−1^ is chosen as the optimal concentration of PEG-nGO, in which the LAMP reaction is not inhibited, but retains enhanced specificity.

To further confirm the beneficial effect of PEG-nGO on LAMP, we analyzed amplified DNAs in both conventional LAMP and the PEG-nGO-based LAMP by gel electrophoresis (Figure 1c). The conventional LAMP without PEG-nGO, multiple ladder bands, and higher molecular weight bands at the top of the gel are observed in both the target and blank samples, showing nonspecifically amplified DNA products. On the contrary, in the presence of PEG-nGO, non-specifically amplified DNAs are not observed in the blank sample, but multiple ladder bands resulting from the target-specific amplified DNAs are observed in the target sample. This gel electrophoresis result is consistent with the real-time fluorescence amplification results, suggesting that PEG-nGO enhances the specificity of LAMP by suppressing non-specific amplicon formation.

**Figure 2:**
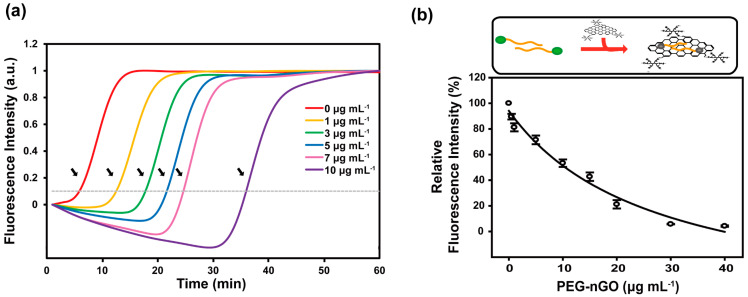
Adsorption of ssDNA LAMP primers on PEG-nGO. (**a**) Real−time fluorescence monitoring of LAMP−amplified products without target under different concentrations of PEG-nGO (0, 1, 3, 5, 7, and 10 μg mL^−1^). (**b**) Schematic illustration showing fluorescence quenching of FAM−labeled ssDNA after adsorption onto PEG-nGO (illustration in box). Fluorescence quenching of FAM−labeled ssDNA with increasing concentration of PEG-nGO. FAM−labeled DNA was incubated with varying PEG-nGO concentration (0, 0.5, 1, 5, 10, 15, 20, 30, and 40 μg mL^−1^) and the fluorescence intensity was measured at an excitation wavelength of 485 nm and an emission wavelength of 535 nm.

To investigate the effect of PEG-nGO on the high concentration of primers, which is responsible for non-specific amplicon generation, we carried out the LAMP without target DNA (no target) under varied concentrations of PEG-nGO (Figure 2a). With the increasing concentration of PEG-nGO, the fluorescence signal lags, with increasing Tt values, suppressing non-specific amplification signals. This result is likely caused by the fact that PEG-nGO enhances the specificity of LAMP by adsorbing excess ssDNA primers onto the PEG-nGO surface, with a decreased chance for non-specific DNA amplification through primer dimerization. We previously reported the adsorption of ssDNA, as well as partially duplex DNA, on the GO surface [27]. The early decrease in SYBR Green fluorescence (up to 30 min) in the LAMP reaction with PEG-nGO is observed due to the probable adsorption of partial duplex DNA amplicons containing SYBR Green fluorophore onto PEG-nGO, which results from the non-specific LAMP reaction. Since the LAMP process continues in the blank sample, due to the non-specific amplification of ssDNA-primed DNA polymerization, duplex DNA amplicons are eventually outnumbered in the end of the LAMP reaction, resulting in fluorescence enhancement after 30 min. Since PEG-nGO retains the properties of GO in terms of ssDNA adsorption, as well as fluorescence quenching, we utilized FAM–DNA for the ssDNA binding test. FAM–DNA (40 mer) that is similar in length to the FIP/BIP primer is employed to verify the adsorption of ssDNA on PEG-nGO. Fluorophore neighboring the surface of GO would be quenched due to a loss in fluorescence emission via fluorescence resonance energy transfer, due to the proximity between GO and fluorophore [27]. As shown in Figure 2b, an apparent decrease in DNA fluorescence is observed with an increasing concentration of PEG-nGO, revealing that ssDNA is readily adsorbed onto the PEG-nGO surface, with approximately 50% fluorescence quenching at 10 μg mL^−1^ of PEG-nGO. This result suggests that PEG-nGO can adsorb the excess amount of ssDNA primers present in the LAMP and decreases possible non-specific DNA amplification.

**Figure 3:**
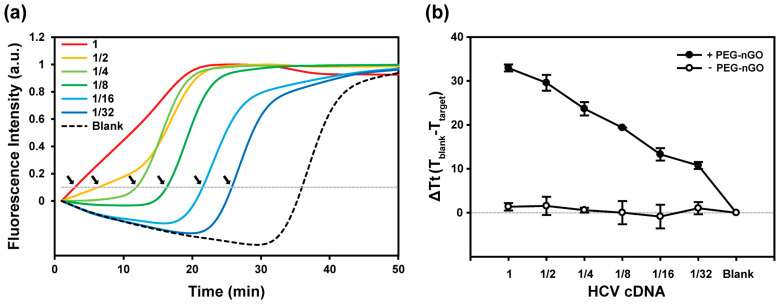
Effect of PEG-nGO on LAMP sensitivity. (**a**) Real−time fluorescence monitoring of PEG-nGO-based LAMP−amplified products with serially diluted concentrations of target cDNA shown with arrows (ranging from 1−fold to 1/32−fold; 1−fold depicts the cDNA transcribed from HCV− positive serum without dilution). (**b**) Comparison of ΔTt (Tt of blank—Tt of target) values of serially diluted target cDNA with or without PEG-nGO. Average ΔTt values are obtained from serially diluted cDNA with or without PEG-nGO, and the error bars display the standard deviation.

Next, to evaluate the effect of PEG-nGO on LAMP sensitivity, serially diluted HCV cDNA, transcribed from HCV RNA, were subject to the LAMP with or without PEG-nGO (Figure 3). In the conventional LAMP without PEG-nGO, fluorescence signals corresponding to Tt from various concentrations of target cDNA are similar to blank samples, resulting in vague detection between target and blank samples with false-positive signals (Figure S2). In contrast, when serially diluted cDNAs are amplified in the PEG-nGO-based LAMP, lagged fluorescence signals with increasing Tt values are obtained as the HCV cDNA samples are serially diluted (Figure 3a). A delayed fluorescence signal with the largest Tt is observed for the blank sample (dashed black line in Figure 3a). The difference between Tt values in the target and blank samples (i.e., ΔTt) of serially diluted HCV cDNA is assessed in the LAMP assay with or without PEG-nGO (Figure 3b). Since the blank and diluted concentrations of the target cDNA show similar Tt values in the LAMP without PEG-nGO, no significant change in ΔTt is observed. On the contrary, Tt is linearly increased with decreasing concentrations of target cDNA, indicating that the dose-response correlation between target DNA concentration and ΔTt values is only observed in the LAMP with PEG-nGO, and not observed in the conventional LAMP. Therefore, the PEG-nGO-based LAMP not only suppresses the fluorescence signal of the blank sample, but also facilitates the amplification of target cDNA in a concentration-dependent manner. These observations demonstrate that the PEG-nGO-based LAMP can detect target viral genes for a wide range of concentrations, which is not attainable in the conventional LAMP.

### 3.3. Detection of HCV-Positive and HCV-Negative Serum Samples by PEG-nGO-Based LAMP

**Figure 4:**
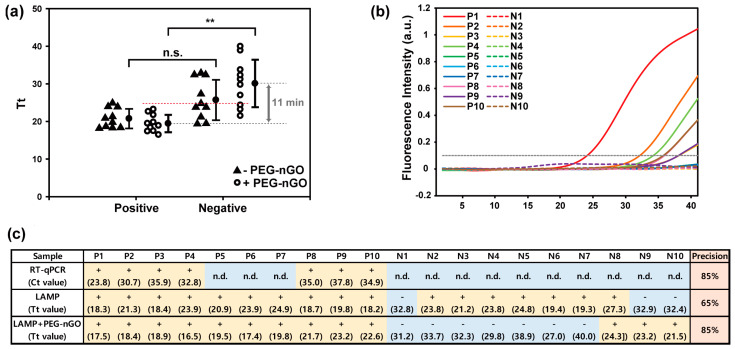
Evaluation of PEG-nGO−based LAMP system for detecting HCV−positive and HCV−negative clinical samples. (**a**) Tt values of 20 HCV clinical samples with 10 positive and 10 negative samples in the presence or absence of PEG-nGO. In conventional LAMP (without PEG-nGO), Tt values are plotted for each positive and negative clinical samples (triangles) and red, dashed line represents the cut−off for decision of positive or negative sample. Tt values are presented as average, and the error bars display the standard deviation, with no statistical significance (n.s., non-specific). In PEG-nGO−based LAMP, Tt values are plotted for each positive and negative clinical samples (circles). Tt values are presented as average, and the error bars display the standard deviation, with statistical significance (**, *p* < 0.01, positive vs. negative). Tt values of both HCV−positive and HCV−negative samples are plotted based on the real−time fluorescence monitoring of LAMP−amplified products. (**b**) Curves of fluorescence change in conventional RT−qPCR analysis with a total of 20 HCV clinical samples including HCV−positive (solid line) and negative samples (dashed line). (**c**) Table shows a comparison of analysis results acquired from the RT−qPCR, conventional LAMP, and PEG-nGO−based LAMP method for 20 HCV clinical samples. All 10 HCV−positive samples are detected as positive in PEG-nGO−based LAMP, while 7 out of 10 samples are found positive in qRT−PCR. Inversely, 3 and 7 out of 10 negative samples are detected as negative in conventional LAMP and PEG-nGO−based LAMP, respectively. Abbreviation: +: detected as positive based on cycle threshold (Ct) or Tt; -: detected as negative based on Ct or Tt; n.d.: non-detected.

Encouraged by the viability of the PEG-nGO-based LAMP in the detection of target DNA with suppressed a background signal, we employed the PEG-nGO-based LAMP for the clinical samples derived from HCV patients; RNAs isolated from 10 HCV-positive (P1-P10) and 10 HCV-negative (N1-N10) serum samples were transcribed into cDNA, which were subject to LAMP assays in the absence or presence of PEG-nGO (Figure 4). First, we optimized the concentration of Bst DNA polymerase in the PEG-nGO-based LAMP assay (Figure S3). We observe that the standard concentration (8 U) of *Bst* DNA polymerase used in the conventional LAMP is not appropriate for the PEG-nGO-based LAMP assays, with indiscriminating Tt values irrespective of positive or negative samples. A significant increase in ΔTt is observed with a decreasing concentration of *Bst* DNA polymerase; ΔTt of 10.9 min and −0.06 min at 2 U and 8 U of *Bst* DNA polymerase, respectively. Thus, 2 U of *Bst* DNA polymerase with the highest ΔTt, is chosen as the optimal enzyme concentration for the detection of HCV-positive and HCV-negative samples.

Next, we performed the PEG-nGO-based LAMP for the 10 HCV-positive and 10 HCV-negative samples, with real-time monitoring of amplicon DNA fluorescence (Figure S4). The fluorescence signals from the LAMP assay display that the fluorescence of the 10 HCV-positive samples increase earlier than those of the 10 HCV-negative samples. Based on the real-time fluorescence traces with Tt values, we grouped Tt values obtained from the HCV-positive and HCV-negative samples in the absence or presence of PEG-nGO (Figure 4a). In the conventional LAMP without PEG-nGO, Tt values of the respective positive and negative samples are found to not be close enough for a significant decision. In contrast, the PEG-nGO-based LAMP assay exhibits statistically discernible Tt values, enough for the right decision on positive and negative samples (difference in averaged Tt between positive group and negative group, 11 min). The diagnostic results obtained from the PEG-nGO-based LAMP assay are compared with the conventional detection method, RT-qPCR (Figure 4b). The real-time fluorescence monitoring of RT-qPCR with HCV-positive and HCV-negative samples shows that only 7 out of 10 HCV-positive samples are identified as HCV-positive with a detectable threshold cycle (Ct, as 10% of the maximum amplified fluorescence intensity) value. However, the PEG-nGO-based LAMP assay identifies all 10 of the HCV-positive samples as positive (Figure 4c). When compared with the conventional LAMP assay, the PEG-nGO-based LAMP displays enhanced precision in the diagnosis of HCV-negative samples, with a significantly reduced frequency of false-positive decisions for the HCV-negative samples by 1.75-fold (7 and 3 false-positive diagnostic decisions out of 10 HCV-negative samples with the LAMP and the LAMP plus PEG-nGO, respectively). Therefore, we suggest that the PEG-nGO-based LAMP can significantly improve the performance of LAMP assays in the diagnosis of clinical samples for the detection of target viral genes within 30 min under isothermal reaction conditions.

## 4. Conclusions

The present work demonstrates the beneficial effects of PEG-nGO on the efficiency and specificity of LAMP assays for viral gene detection. LAMP assays are quick and simple, but often tend to generate false-positive signals. Herein, we demonstrate that PEG-nGO at an optimal concentration notably improves the LAMP specificity, by adsorbing excess DNA primers and, thus, suppressing non-specific gene amplification caused mainly by primer dimerization. The PEG-nGO-based LAMP is, thus, able to distinguish target DNA samples from the blank samples with augmented ΔTt values of 0.15 and 33.3 in the absence or presence of PEG-nGO, respectively, making it more sensitive than the conventional LAMP assay. The PEG-nGO-based LAMP was applied for the diagnostic detection of 10 HCV-positive and 10 HCV-negative samples derived from human serum samples. The PEG-nGO-based LAMP successfully diagnoses all 10 HCV-positive samples, while only 7 samples are detected as HCV-positive in qRT-PCR. Additionally, the PEG-nGO-based LAMP significantly enhances the detection precision for false-positive decision by 1.75-fold, as compared to the conventional LAMP. Thus, PEG-nGO can significantly improve the performance of LAMP assays by facilitating specific amplification of target DNA, with a decrease in background signal through the adsorption of excess ssDNA primers in the LAMP reaction. We suggest that PEG-nGO-based LAMP has a practical applicability in target gene detection, with high sensitivity and specificity.

## Data Availability

Not applicable.

## Supplementary Materials

The following supporting information can be downloaded at: https://www.mdpi.com/article/10.3390/bios12080661/s1. Table S1: DNA primers for LAMP and RT-qPCR used in this study.; Figure S1: Tt values for target and blank in the presence of various PEG-nGO concentrations; Figure S2: Real-time fluorescence monitoring of conventional LAMP reactions (- PEG-nGO) with serially diluted target cDNA concentrations.; Figure S3: Tt values of positive and negative HCV clinical samples in the presence of various amounts of *Bst* DNA polymerase with PEG-nGO.; Figure S4: Real-time fluorescence monitoring of LAMP reactions for 20 clinical samples with 10 positive (solid line) and 10 negative (dashed line) in the presence of PEG-nGO.
