# Supplementary material for: Enhanced Specificity in Loop-Mediated Isothermal Amplification with Poly(ethylene glycol)-Engrafted Graphene Oxide for Detection of Viral Genes
Source: Biosensors (Basel). 2022 Aug 20;12(8):661. doi: 10.3390/bios12080661 (PMC9405610; doi:10.3390/bios12080661)
Supplement: Supplementary file 1 [file biosensors-12-00661-s001.zip › biosensors-1864577-supplementary.pdf]

biosensors

MDPI

Supplementary material: Enhanced Specificity in Loop-Mediated Isothermal Amplification with Poly(ethylene glycol)-Engrafted Graphene Oxide for Detection of Viral Genes

Jamin Ku [1,†], Khushbu Chauhan [1,†], Sang-Hyun Hwang [2], Yong-Joo Jeong [3,*] and Dong-Eun Kim [1,*]

[1] Department of Bioscience and Biotechnology, Konkuk University, 120 Neungdong-ro, Gwangjin-gu, Seoul 05902, Korea
[2] Department of Laboratory Medicine, Asan Medical Center, University of Ulsan College of Medicine, Seoul 05505, Korea
[3] School of Applied Chemistry, Kookmin University, Seoul 02707, Korea
* Correspondence: jeongyj@kookmin.ac.kr (Y.-J.J.); kimde@konkuk.ac.kr (D.-E.K.)
† These authors contributed equally to this work.

Table S1. DNA primers for LAMP and RT-qPCR used in this study.

Method	Name	Sequence (5′ → 3′)	Size (nt)
PEG-nGO-based/ conventional LAMP	F3	CAC TCC CCT GTG AGG AAC T	19
	B3	ACT ACT CGG CTA GCA GTC TT	20
	FIP	TGG AGG CTG CAC GAC ACT CAA CTG TCT TCA CGC AGA AAG C	40
	BIP	CTG CGG AAC CGG TGA GTA CAC AAT CTC CAG GCA TTG AGC G	40
	LF	ACT AAC GCC ATG GCT AGA C	19
	LB	GAC GAC CGG GTC CTT TC	17
RT-qPCR	Forward Primer	TAA TAC GAC TCA CTA TAG GAA TCA CTC CCC TGT GAG GAA CT	41
	Reverse Primer	TGC ACG GTC TAC GAG AC	17

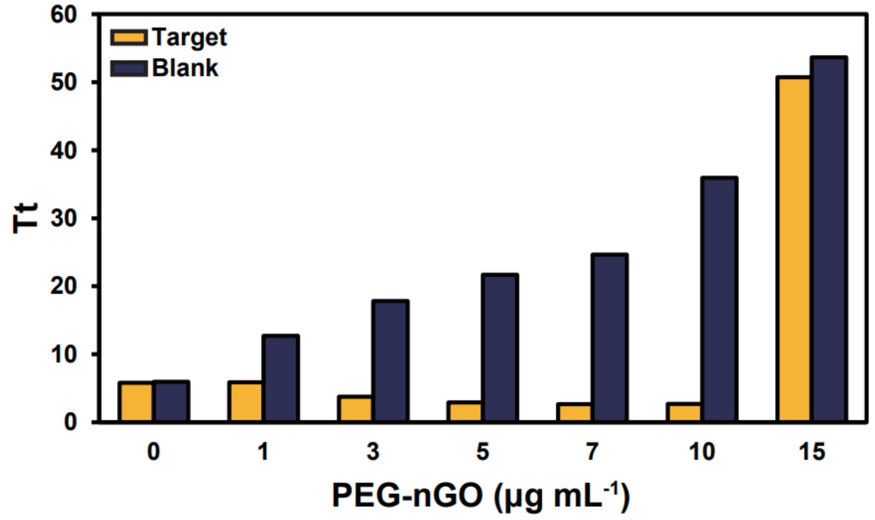

Figure S1. Tt values for target and blank in the presence of various PEG-nGO concentrations (0, 1, 3, 5, 7, 10, and 15 µg mL^{-1}). Tt values were plotted for various concentrations of PEG-nGO (0-15 µg mL^{-1}) based on the real-time fluorescence monitoring of LAMP.

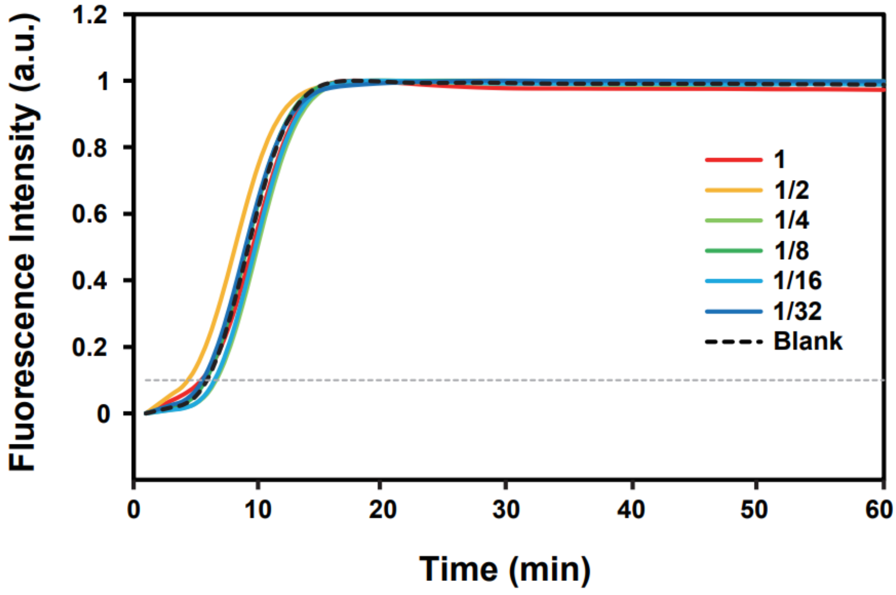

Figure S2. Real-time fluorescence monitoring of classic LAMP reactions (- PEG-nGO) with serially diluted target cDNA concentrations (ranging from 1-fold to 1/32-fold; 1-fold depicts the cDNA transcribed from HCV-positive serum without dilution).

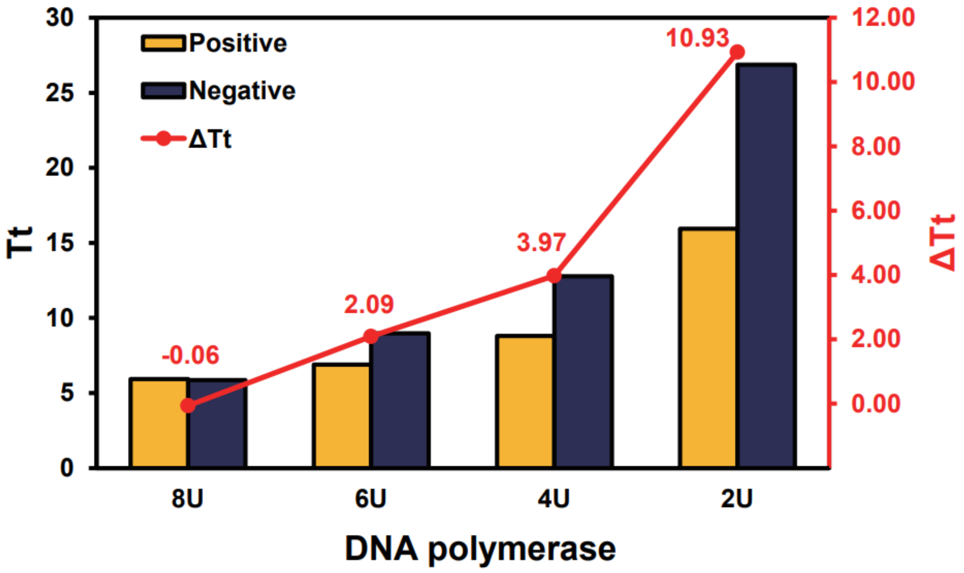

Figure S3. Tt values of positive and negative HCV clinical samples in the presence of various amounts of *Bst* DNA polymerase (2, 4, 6, 8 U) with PEG-nGO. Tt values were plotted based on real time fluorescence monitoring of various amounts of *Bst* DNA polymerase with PEG-nGO.

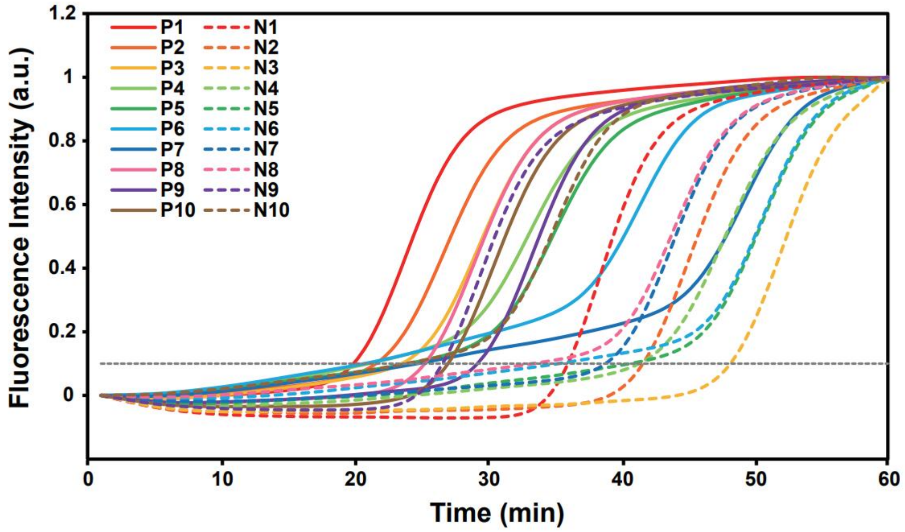

Figure S4. Real-time fluorescence monitoring of LAMP reactions for 20 clinical samples with 10 positive (solid line) and 10 negative (dashed line) in the presence of PEG-nGO.
